# Understanding the Effects of Anesthesia on Cortical Electrophysiological Recordings: A Scoping Review

**DOI:** 10.3390/ijms22031286

**Published:** 2021-01-28

**Authors:** Vincenzo Sorrenti, Claudia Cecchetto, Marta Maschietto, Stefano Fortinguerra, Alessandro Buriani, Stefano Vassanelli

**Affiliations:** 1 Department of Pharmaceutical & Pharmacological Sciences, University of Padova, 35131 Padova, Italy; 2 Maria Paola Belloni Center for Personalized Medicine, Data Medica Group (Synlab Limited), 35100 Padova, Italy; alessandro.buriani@gmail.com; 3 Optical Neuroimaging Unit, Okinawa Institute of Science and Technology Graduate University, Okinawa 904-0495, Japan; claudia.cecchetto@unipd.it; 4 Department of Biomedical Sciences, Section of Physiology, University of Padova, via F. Marzolo 3, 35131 Padova, Italy; marta.maschietto@unipd.it; 5 Padua Neuroscience Center, University of Padova, via Orus 2/B, 35131 Padova, Italy; 6 IRCCS SDN, 80143 Napoli, Italy; stefano.fortinguerra@gmail.com

**Keywords:** anesthesia, sevoflurane, propofol, ketamine, cortical recordings, electrophysiology

## Abstract

General anesthesia in animal experiments is an ethical must and is required for all the procedures that are likely to cause more than slight or momentary pain. As anesthetics are known to deeply affect experimental findings, including electrophysiological recordings of brain activity, understanding their mechanism of action is of paramount importance. It is widely recognized that the depth and type of anesthesia introduce significant bias in electrophysiological measurements by affecting the shape of both spontaneous and evoked signals, e.g., modifying their latency and relative amplitude. Therefore, for a given experimental protocol, it is relevant to identify the appropriate anesthetic, to minimize the impact on neuronal circuits and related signals under investigation. This review focuses on the effect of different anesthetics on cortical electrical recordings, examining their molecular mechanisms of action, their influence on neuronal microcircuits and, consequently, their impact on cortical measurements.

## 1. Introduction

Anesthesia or anaesthesia (from Greek “without sensation”) is a state of controlled, temporary loss of sensation or awareness induced for medical purposes like surgical or painful procedures. It may include some or all of the following: analgesia (relief from or prevention of pain), immobility (muscle relaxation), amnesia (loss of memory), and unconsciousness. [1]. Anesthetics are capable of producing anesthesia from in species from *C. Elegans* to humans. This action that goes from the two extremes of the animal world explains how anesthetic targets are highly conserved in the evolutionary scale. In fact, the contribution of animal experiments has played a relevant role in the evolution of anesthesia. However, several drugs and techniques not used in humans are being used in veterinary anesthesia and in some animals anesthetic effect can be quite challenging because of their variable anatomies, lifestyles and aggressive behavior [2,3].

General anesthesia in animal experiments is required for all the procedures likely to cause more than slight or momentary pain or discomfort in animals [4]. Anesthesia can also be used as a restraint method for procedures that would cause excessive stress to the animal and expose the operator to potential hazards [5] and, beyond the duration of general anesthesia, pain relief should also be provided postoperatively [6]. If animals undergo survival surgery, they need to recover quickly and without suffering, which is crucial for animal wellbeing and scientific research quality [7]. General anesthesia can be achieved with injection or inhalation of substances that induce a reversible state of unconsciousness [1]. Due to the different intervention priorities, the types of anesthetics commonly used in human beings and laboratory animals vary considerably (see Table 1). Electrophysiological measurements of brain activity, as well as other experimental parameters, are influenced by anesthesia in an anesthetic-dependent manner [8]. The use of general anesthetics in particular can impact considerably the findings as they act with specific tropism on different brain regions and alter various neuronal functions [9]. Moreover, effects depend on the dosage and the way of administration (inhalation or parenteral route) [10,11] (Box 1). Understanding the influence of the commonly used anesthetics on electrophysiological measurements of the brain cortex is a crucial topic within the neuroscience community. This review will focus on the effect of different anesthetics on cortical recordings, examining their mechanisms of action, the influence they exert on neuronal circuits, and, consequently, their impact on measured signals. For example, the depth and type of anesthesia are known to introduce significant bias in electrophysiological recordings in the somatosensory cortex, affecting the shape of evoked and spontaneous responses, and modifying the latency and amplitude of measured events. Thus, anesthetics are reviewed in the attempt to help researchers to identify the type of anesthetic that has the minimum impact on neuronal recordings within the specific experimental setting, including the type of electrophysiological signal and brain area under consideration, and the neuronal microcircuits involved.

Box 1 **Anesthetic bioavailability.**

The choice of an anesthetic strongly depends on its chemical-physical properties, the route of administration (injected or inhaled), and its bioavailability and distribution kinetics in the body [12,13]. Many drugs are administered via injection for induction and maintenance of anesthesia, and their action can be prolonged by combination with inhalation agents. The lipophilic nature of anesthetics allows them to act on the central nervous system (CNS), whose structure is rich in lipids. Every administered dose follows a distribution process in the body with a preferential brain tropism and with a meaningful fraction that distributes in the blood, muscles, and fats at different speeds. At the same time, a residual dose fraction is metabolized and excreted irreversibly [14,15]. For in vivo anesthetic procedures, which can last from a few minutes to hours, achieving a steady-state strongly depends on the strategy of drug delivery and can require additional doses after the induction or a continuous and constant delivery of the anesthetic. By using a syringe driver for intravenous infusion anesthesia or when performing anesthesia using volatile anesthetics a steady state of anesthesia can be maintained for hours [16]. The effects related to a single dose of anesthetic change over time due to the anesthetic distribution between body compartments and are usually correlated to the anesthetic concentration at the site of action [12,17]. The physical-chemical nature of the anesthetic significantly affects its bioavailability. Intravenous anesthetics produce anesthesia rapidly and are generally used to induce and maintain anesthesia for short operative procedures (<30 min). However, inhalational anesthetics are often only used to prolong the anesthetic state as partial anesthetic pressure of an inhalation agent is reached slowly [18,19]. Anesthetic drugs administered intravenously are particularly suitable to meet the first requirement of anesthetic management, i.e., the rapid induction of the state of unconsciousness [19]. These compounds generally induce anesthesia within seconds of administration because of their lipophilic nature, and consequently spread rapidly across all biological membranes, including the blood-brain barrier, quickly reaching high brain concentrations [20]. In addition, since brain tissue receives a large percentage of cardiac output, a considerable proportion of an intravenously administered anesthetic is distributed to the CNS. Anesthetic tropism for the CNS does not persist for a long time because balancing forces tend to redistribute the anesthetic concentration with other less-perfused organs. Therefore, after the first phase of distribution in the CNS, the anesthetic is redistributed from the blood to less-perfused tissues and partially metabolized and excreted, leading to reduced plasma and brain concentration [21]. The half-life defines the initial redistribution rate following a single intravenous drug administration, and is generally about few minutes for most anesthetics [22] depending on the type and dosage, with an average return to consciousness from 15 to 30 min post-injection [23]. Propofol, as an example, acts rapidly and has a short recovery time. Rapid onset of anesthesia (50 s) is achieved, and if no other drug is administered, recovery will take place in 4 to 8 min. The recovery is associated with redistribution of the drug and rapid metabolism to glucuronide and sulfate conjugates in the liver and extrahepatic tissues, such as the intestine and kidney.

Inhalational anesthetics are preferably used to maintain anesthesia for surgical procedures longer than 30 min. Brain anesthetic concentration is reached more slowly than with intravenous drugs. However, once anesthesia is achieved, it is maintained over time by controlling the gas supply rate [18,24]. The recovery time from inhaled anesthetics in long-lasting surgical procedures is more rapid than intravenous ones, thanks to pulmonary anesthetic elimination, which is not dependent on the slow metabolic clearance rate of tissues [12]. Therefore, inhalation drugs meet the requirement for a relatively rapid return of the animal’s psychomotor skills if necessary. The pharmacokinetic factors influencing inhalational anesthetic bioavailability involve concentration gradients and partial pressure gradients according to Henry’s law. The final anesthetic concentration in a tissue is a function of the partial pressure and affinity for that tissue. The brain, having a high blood flow per unit of mass, balances first with the alveolar tension of the anesthetic. Tissues with a lower blood flow take a longer time and accumulate anesthetic gases during the anesthesia maintenance phase [25].

Anesthetic solubility is another critical factor for anesthesia induction to establish a level of unconsciousness suitable for surgery [12,26]. Anesthetics with limited plasma solubility and a low absorption rate (e.g., sevoflurane and desflurane) balance quickly with the tissues, unlike highly soluble plasma anesthetics (e.g., methoxyflurane), where a higher initial absorption delays the increase in alveolar tension at the inspired level and the gas balance with the brain into the plasma from the alveoli [25]. Compared to “older” inhalational agents such as isoflurane or halothane, sevoflurane has low solubility in the blood. This results in a more rapid uptake and induction, improved control of anesthesia depth, and faster elimination and recovery.

Similarly to the uptake, the elimination of a volatile anesthetic is related to its solubility in blood and tissues. Between 95 and 98% of the amount of sevoflurane taken up is eliminated through the lungs. The driving force is the difference in partial pressures between the inspired gas mix and the pulmonary capillary blood. As only 2 to 5% of the absorbed dose of sevoflurane is metabolized, metabolic clearance can be ignored for the pharmacokinetics [27]. Finally, the rate of lung perfusion (which is equivalent to cardiac output in healthy animals) also influences the induction of anesthesia [28]. Since more blood will pass through the pulmonary capillary bed when the cardiac output is high, a greater total transfer of any anesthetic agent across the alveolus will occur in these conditions. As a consequence, tissues normally receiving a smaller proportion of the total cardiac output receive a greater amount when cardiac output is high and accumulate a larger proportion of the anesthetic crossing the alveolar membrane. Ultimately, greater uptake will slow the rate of rising of the alveolar tension–time curve, and anesthetic induction with an individual agent may be slower when the cardiac output and perfusion of the lung are high. In low cardiac output states, the opposite is true. The uptake rate will be lower, and the alveolar tension will rise toward the inspired tension more quickly. To minimize cardiac output effect on the rate of induction of anesthesia, agents of lower solubility would be preferred clinically [18]

## 2. Understanding the Mechanism of General Anesthesia: How Far Are We?

The induction of general anesthesia is the final result of complex alterations of the normal state of consciousness that can be induced in a multiple and diversified manner by different classes of anesthetics. In general, besides causing unconsciousness anesthetics should block the brain’s ability to integrate information [25,29,30]. Amnesia, hypnosis and immobility are essential components that can involve a variety of distinct molecular targets with plenty of opportunities for improvement of anesthesia strategies [31]. Before an anesthetic action at the molecular level becomes manifest at the systemic level a complex integration of signals occurs within the central nervous system fueling the debate about what molecular targets and mechanisms are more relevant. Despite the fact that the mechanisms of anesthetics remain partially elusive, novel molecular targets are continuously emerging [30,31,32].

It is therefore important to analyze the mechanisms of action of anesthetics at different levels, from receptors, enzymes, and ion channels to synapses and, finally, on neuronal circuits.

General anesthesia is associated with a functional uncoupling of neuronal excitation leading to impairment of top-down signaling, thus causing both regional suppression of brain activity and disruption of large-scale neuronal networks. This determines neuronal transmission inefficiency and networks de-synchronization, enhancing modularity and the dissociation of functions both within an organism and between the organism and its surroundings [33,34].

One common feature is that anesthesia causes CNS perturbations by increasing neuronal inhibition or decreasing neuronal excitation or both, e.g., by altering Gamma-Aminobutyric Acid (GABA)-ergic transmission [35]. During this process, GABAergic synaptic responses are enhanced, GABA-activated Cl– currents are potentiated, and N-Methyl-D-aspartic Acid (NMDA) and α-Amino-3-hydroxy-5-Methyl-4-isoxazolePropionic Acid (AMPA) inducing excitatory postsynaptic current (EPSC) is suppressed [36,37,38].

Not all the brain regions are affected to the same extent by general anesthetics. Different anesthetics act on different pharmacological targets, or with different affinity for the same targets. Thus, they alter a number of neuronal circuits and multiple brain targets in a differentiated manner [29]. Moreover, anesthetics differ extensively in their chemical, physical, and pharmacological properties, greatly varying in size and chemically active groups, in effect forming the most heterogeneous pharmacological class [14]. Recent techniques like photolabeling and substituted cysteine modification- protection (SCAMP) are being applied to a number of important questions related to mechanisms of general anesthesia. Structure activity relationships acquired during photolabel development have also proven useful in developing novel clinical anesthetics [39].

There are many molecular targets of anesthetics within the central nervous system [38,40]. In general, the chemical structures of anesthetics suggest that their macromolecular binding sites are likely hydrophobic, small, but with a certain degree of polarizability, which are characteristic features of internal cavities or structural pockets [39]. Collectively it has been evaluated that over 300 proteins are potentially anesthetic binding targets. Although this endows anesthetics with a high level of target promiscuity, the degree of protein selectivity for such general sites is surprisingly high, possibly due to differences in the implication of van der Waals forces and the presence of an H-bond donor/acceptor, depending on the specific type of drug. Anesthetic modulatory sites have been subclassified into intra- and inter-subunit, and pore-lining binding sites. Structural, biochemical, and functional observations indicate that anesthetic agents bind or act at multiple modulatory sites rather than acting on a unitary target, ultimately leading to mixtures of inhibiting and potentiating effects to produce the final collective outcome [40]. In this pharmacological context, where multiple targets can be affected, their functional alignment plays a central role in drug effectiveness: functional alignment of the collective effect produced by ligand occupancy of different sites leads to pharmacological additivity or synergy, while functional non- or partial alignment from variably occupied sites can produce different effects, a phenomenon that might underlie the biphasic activity commonly observed with anesthetics [41]. While in the past much attention has focused on ion channels (GABAA, NMDA, etc.), other proteins have been found to be sensitive to anesthetics as well [29]: currently under investigation and definitely of interest are, for example, metabotropic receptors including muscarinic acetylcholine (ACh) M(1), metabotropic type 5 glutamate, 5-hydroxytryptamine (5-HT) type 2A, and substance P receptors [42] which modulate synaptic transmission and partly bind the same ligands as ligand-gated ion channel receptors. Pavel et al. recently demonstrated that inhaled anesthetics robustly activate TWIK-related K^+^ channels (TREK-1) and reversibly induce loss of consciousness [43]. Other proteins affected by anesthetics are protein pumps, gap junctions in electrical synapses, protein kinases, and phosphatases, as well as adrenergic receptors, prostanoid receptors, motility proteins, SNARE (soluble N–ethylmaleimide sensitive factor attachment protein receptor) proteins, and fatty acid amide hydrolase (FAAH) [29,38,44]. Recently, a pilot investigational study on mouse brain reported that anesthetics with differing synaptic receptor mechanisms, all induced changes in tissue oxygen handling and cortical network activity, through a common inhibitory effect on mitochondrial functions [45]. The anesthetic sensitivities of only a few proteins have been investigated, if compared with the estimated number of at least 12,000 different membrane proteins, of which ion channels are only a small fraction. The list of known anesthetic molecular targets [29] is steadily increasing as ongoing research is continuously revealing new targets. Understanding the relationship between actions at the molecular level and the behavioral effects is, therefore, the ultimate goal of molecular studies of anesthesia.

## 3. Effects of Different Anesthetics on Cortical Electrophysiological Recordings

In vivo electrophysiological techniques provide an objective measure of neurological function. Most of them are carried out under anesthesia to ensure the animal’s unconsciousness and the absence of pain. Neuronal circuits involved in anesthesia are largely intricate and characterized by a plethora of neurotransmitters, ionic channels and molecules that contribute to generate the overall state of anesthesia. Therefore, it is very intuitive that the use of anesthesia can induce important changes in cortical brain activity, which vary between different anesthetics because of their specific mechanisms of action. This, in turn, may influence the electrophysiological recordings, for which various anesthetics having different mechanisms of action may be used. The following section summarizes the effects of most commonly used anesthetics.

### 3.1. Injectable Anesthetics

In complex surgical procedures on humans, which require a deep anesthesia level, injectable i.v. anesthetics are usually used for both induction and maintenance; in some cases, injectable anesthetics are provided for induction and then substituted with inhalation of a different compound for maintenance [46]. In laboratory animals, injectable anesthetics are commonly used for both the induction and the maintenance of the anesthesia throughout the entire surgical and experimental sessions [19]. The initial dose, which provides induction, is followed by additional doses at a reduced concentration. The time intervals between the induction and the additional doses depend on the particular anesthetic used, but in general the first additional dose is provided in 10–30 min after the induction and is then followed by other doses at longer and regular time intervals, thus allowing to maintain a constant anesthesia level. Injectable anesthetics, which belong to different pharmacological classes, have different mechanisms of action: urethane, for instance, modulates neurotransmission by acting on several targets; others, on the contrary, are more selective for specific receptors [19].

#### 3.1.1. Urethane

Urethane (ethyl carbamate) is a water-soluble compound which is widely used as an anesthetic in neurophysiological experiments on animals. Being a carcinogen, its use as a human anesthetic is instead precluded. It is the preferred anesthetic for investigations of neuronal function in visual, somatosensory and hippocampal cortical regions, because it does not appear to alter or depress sensory evoked responses or neuronal discharge patterns to the same degree as volatile or barbiturate anesthetics [47,48,49]. The advantages of urethane in animal anesthesia are that it can be administered by several parenteral routes, it produces a long-lasting steady level of surgical anesthesia, and has minimal effects on autonomic and cardiovascular systems. It is assumed that animals anesthetized with urethane present similar physiological and pharmacological behaviors to those observed in unanesthetized animals [50]. Despite the usefulness of urethane in animal research and in contrast to other injectable anesthetics, there is limited data concerning its molecular actions, and its effects on GABAergic neurotransmission are not clear [47]. Bowery and Dray, for instance, reported that urethane reversed the antagonistic effect of bicuculline on GABA-induced depolarization in the isolated rat superior cervical ganglion [50]. However, other investigations have indicated that urethane produces only minimal enhancement of GABAergic neurotransmission at a clinical concentration. In particular, Sceniak et al. found that urethane strongly depressed action potential discharge of cortical neurons in vitro, in response to depolarizing current, together with a significant decrease in membrane resistance. These effects were related to a selective activation of a Ba^2+^-sensitive K^+^ leak conductance without altering excitatory glutamate-mediated or inhibitory (GABA_A_- or GABA_B_-mediated) synaptic transmission. These results are consistent with the other reported observations during urethane anesthesia where urethane exerted minimal disruption of signal transmission in the neocortex [51,52]. Therefore, it is conceivable that there might be other targets for urethane: actually, it has been demonstrated that urethane potentiated the functions of neuronal nicotinic acetylcholine, gamma-aminobutyric acid(A), and glycine receptors, and it inhibited N-methyl-D-aspartate and alpha-amino-3-hydroxy-5-methyl-4-isoxazole propionic acid receptors in a concentration-dependent manner. At concentrations close to anesthetic 50% effective concentration, urethane had modest effects on all channels tested, suggesting the lack of a single predominant target for its action. This may account for its usefulness as a veterinary anesthetic. However, a large concentration of urethane exerts marked effects on all channels [47,50,51].

Urethane is frequently used in recordings of spontaneous and/or evoked activity in rodents’ brains (see Box 2 for an example of evoked activity). In P28–P42 Wistar rats anesthetized with i.p. injection of 1.5 g/kg urethane, followed by additional 10% doses providing deep anesthesia levels, the speed of activity propagation across the barrel cortex surface was faster for the whisker stimulation-evoked than for the spontaneous events [53]. Long term electroencephalography (EEG) recordings from frontal and posterior neocortex and dorsal hippocampus in adult Wistar rats anesthetized by slow intravenous administration of urethane (1.75 g/kg body mass) revealed similarities with the alternate pattern of REM and nREM states occurring during sleep. Periods of low amplitude and fast activity in the neocortex, which are typical of REM phase, and concomitant to theta activity in the hippocampus, alternated to high amplitude and slow oscillatory activity (equal to nREM) in both neocortex and hippocampus. Supplemental doses of urethane only decreased the relative amount of time spent in the activated state, without altering the rhythmicity of state alternations. Again, similarly to natural sleep, consistent increases in both heart and respiration rates were concomitant with transitions from nREM to REM states [54]. The features of evoked responses in the cortex of rodents anesthetized with urethane were shown to be different from those of other anesthetics. For instance, evoked response potentials (ERPs) were recorded by a 25-channels electrode array over the somatosensory cortex during whiskers stimulation of adult Sprague-Dawley rats, and their main components (gamma: 25–45 Hz, fast oscillations FO: 200–400 Hz, very fast oscillations VFO: 400–600 Hz) were compared under different anesthetics regimens. Urethane, administered intraperitoneally at the dose of 1.5 to 2 g/kg body mass, induced the major increase of the latency of ERPs with respect to what occurred with ketamine-xylazine, isoflurane, propofol and pentobarbital sodium. Urethane also strongly reduced the response amplitude for FO and VFO, even though the main effect in overall amplitude decrease was detected under isoflurane anesthesia [55]. Applying multisite optical voltage sensitive dye (VSD) recordings on the somatosensory cortex under hindpaw electrical stimulation, the effects of urethane administered i.p. were compared to urethane + alpha-chloralose in adult Sprague-Dawley rats: no significant differences in the amplitude of neural response between the two anesthetic conditions were observed, but under α-chloralose anesthesia the propagation velocity of neural excitation was larger, while the duration was shorter. These differences were mainly attributed to the presence of α-chloralose, whose enhancement of GABA-induced currents appears to be stronger than urethane depression of glutamatergic transmission [56]. Despite its effect on the evoked responses, the intraperitoneal delivery of urethane (1.6–2 g/kg, with additional 10–20% of the initial dose) is commonly used in experimental electrophysiology in rodents (e.g., by Unichenko and collaborators to compare wild type and the autism model Nlgn4-KO mice neuronal activity by recording local field potentials (LFPs) from S1 barrel cortex with a 8-shank 128-channel electrode under single whisker paired-pulse stimulations in the rostro-caudal direction [57]). Concerning the intraperitoneal route of administration, different groups reported a dependence of the brain activity on the dose of urethane: the dose was found to influence not only the rat barrel cortex spontaneous activity in the infragranular layers, but also its relationship with whisker deflection-evoked neuronal activity recorded with a multichannel array under three different anesthesia levels. Increasing the depth of anesthesia from light (induction 1.5 g/kg body weight i.p.) to moderate (induction followed by one 20% supplementary dose after 1 h) to deep (induction followed by two 20% additional doses after every hour), urethane decreased both burst and firing rates in the spontaneous activity, and concomitantly increased the burst duration and the number of spikes occurring within bursts. Under light urethane anesthesia, responses to the principal whisker and to surrounding whiskers were robust; with increasing depth of anesthesia, large populations of cortical neurons bounded together in cyclic waves of hyperpolarization and depolarization, thus becoming less responsive to sensory inputs from whisker stimulation. At deeper levels of anesthesia, cortical neurons responded exclusively to the inputs arising from the thalamic ventral posterior-medial nucleus (VPM), therefore losing their responsiveness to all whiskers except the principal one [58]. Devonshire and collaborators recorded somatosensory evoked potentials (SEPs) and VSD imaging data from the primary somatosensory cortex surface of adult Wistar Han rats under single whisker deflection of 2 mm in the caudal direction: a comparison of the results revealed a reduction of the amplitude of the evoked responses on both SEPs and VSDI, which was dependent on the dose of i.p. urethane. This effect was also related to the frequency of stimulations, being much more marked for high frequency (10 Hz) respect to low frequency (2 Hz). Conversely, no significant changes among anesthetic levels were observed on latency and shape of the responses [59].

#### 3.1.2. Ketamine

Ketamine is an arylcyclohexylamine, a congener of phencyclidine. It is usually supplied as a racemic mixture, but its S-enantiomer alone, known as esketamine, is frequently used in veterinary medicine as it is more powerful and has fewer side effects. Ketamine is a non-barbiturate, fast-acting drug with general anesthetic action, mainly for parenteral use. When administered to the experimental animal via the venous route, it produces a dissociative anesthesia. In humans, intravenous administration of 1 mg/kg causes analgesia and anesthesia in 30 s; the anesthetic state is then maintained for 3 to 10 min. Blood pressure and heart rate increase, while breathing is initially depressed and bronchodilation occurs [60]. The analgesic and anesthetic action of ketamine has been linked to the NMDA receptor blocking activity [61]. Some studies on murine models demonstrated that its pharmacological activity is not determined by the inhibition of the NMDA receptor, but, rather, by a sustained activation of a different glutamate receptor, the AMPA receptor, suggested to be exerted by its metabolite (2R, 6R) -hydroxynorketamine [62]. High-dose ketamine is also capable of binding to μ-type opiate receptors [63]. Ketamine interacts with muscarinic receptors, affecting the descending pathways of monoamine pain and with voltage-dependent calcium channels. It basically causes dissociative anesthesia, with depression of the thalamo-cortical system and activation of the limbic system. The blockage of painful sensitivity occurs at the integumentary and non-visceral level. The osteotendinous, ciliary, corneal, photomotor, pharyngeal and laryngeal reflexes, instead, are preserved; pupillary dilation and vertical or horizontal nystagmus occur [61]. Ketamine has an anticonvulsant action in both animals and humans. The drug determines an increase in brain oxygen consumption, with an increase in brain flow and a reduction in cerebral vascular resistance. In addition, the cerebrospinal fluid pressure increases. As a result, respiratory rate (except for small rodents), sympathetic tone and airway secretions increase. The drug also activates the monoamine oxidases (MAOs) and causes an enzymatic cyclization of the catecholamines, with an increase in toxic cyclic derivatives. These changes are the cause of the hallucinatory phenomena that can occur upon awakening [64].

Since it is capable of suppressing respiration much less than most of the other available anesthetics, ketamine is still widely used in the medical field. However, mainly due to the hallucinations it can cause, it is not generally used as a primary anesthetic, although it is considered the anesthetic of choice when reliable ventilation equipment is not available. Ketamine is also one of the few anesthetics that can be administered orally, intravenously and intramuscularly [65].

In animals, particularly in rats, the effect of intraperitoneal injection of ketamine alone was compared to the awake condition. In particular, a chronic implant of a high-density micro-electrocorticography (micro-ECoG) grid was realized over the whisker somatosensory cortex: the LFPs evoked by the snout skin stimulation during anesthesia were much larger and lasted much longer than those during wakefulness; however, LFPs propagated with a similar topography, i.e., from the site showing the the response onset outwards in all directions with decreasing amplitude, thus suggesting that ketamine only weakly affected the degree of spreading of neuronal activity [66,67].

#### 3.1.3. Ketamine-Xylazine/Medetomidine

Although sometimes it is used alone, ketamine is usually combined with alpha2 adrenoceptor (α2-AR) agonists like xylazine or medetomidine to provide analgesia. Xylazine is an analogue of clonidine which reduces Norepinephrine (NE) and Dopamine (DA) release in the CNS, acting on the NE inhibitory network. Since NE elicits thalamo-cortical and intracortical excitatory inputs, the use of xylazine could lead to some degree of interference in the electrophysiological measurements and a delay in response onset [68,69,70].

The effects of light and deep anesthesia levels obtained with an induction mixture of ketamine (100 mg/kg) and xylazine (5 mg/kg) i.p. were characterized in adult Sprague-Dawley rats [71]. Intracellular recordings in layers II/III of the piriform cortex revealed high-amplitude, low-frequency oscillations of membrane potential, which were phase-related with respiration, during deep anesthesia (that is, 5–30 min after a 30% additional dose). Under light anesthesia (that is, more than 40 min after an additional dose) low-amplitude, high-frequency oscillations did not show any phase-relation with the respiratory band and were concomitant with small amplitude whisking and forepaw reflexes. The simultaneous extracellular field potentials recordings in the superficial layer of the piriform cortex and in the granule cell layer of the olfactory bulb revealed large amplitude fluctuations correlated with respiration under deep anesthesia. In case of light anesthesia, instead, small amplitude fluctuations were recorded in the piriform cortex, while only the olfactory bulb maintained a more oscillatory pattern and a clear correlation with the respiration. The anesthesia levels were shown to influence also the frequency distributions of activity in membrane potential, piriform cortex and olfactory bulb field potentials: deep anesthesia was associated with increased activity in the low-frequency ranges, while all the three types of recordings had larger gamma band frequency components in the light anesthetic state. Ketamine-xylazine level-dependent results were observed in other cortical areas and with a different administration route: Tandon and collaborators reported that with light intramuscular anesthesia, defined by the presence of mild spontaneous whisking, pinch withdrawal, eyelid reflexes and high heart rates (201–257 beats⁄min), a peak at 5–7 Hz was prominent in the power spectrum of the electrocorticogram (ECoG) of the parietal cortex in adult rats; with deep i.m. anesthesia (no reflex and no spontaneous whisking, with 192–216 beats/min heart rates), instead, the ECoG main peak was at 1–2 Hz. Furthermore, intracortical microstimulation in the M1 motor cortex with light anesthesia induced retraction of the whole whisker pad or of a group of adjacent whiskers; at deep anesthesia, instead, no whisker movements could be evoked in any of the rats, even when current amplitudes as high as 150 uA were tested [72]. Micro-EcoG recordings of motor cortex spontaneous activity revealed differences between Sprague-Dawley rats anesthetized with ketamine-xylazine (55 mg/kg and 12 mg/kg i.p., respectively) and awake-quiet rats: the power spectrum under anesthesia was significantly higher below 50 Hz, with respect to the awake group; anesthesia slightly decreased the motor cortex coherence for frequencies lower than 200 Hz, and the signals became more correlated to each other [73]. Multielectrode-array recordings from trunk and hindlimb regions of the primary somatosensory cortex of rats anesthetized with ketamine-xylazine i.p. revealed differences between spontaneous and stimulus-evoked multi-unit activity (MUA) and LFPs slow-wave activity. The onset of evoked response was mainly detected in the thalamorecipient layer IV, whereas the onset of spontaneously occurring up-states was found most frequently in layer V. Thus, thalamocortical pathways might be activated for longer time scales upon stimulation, resulting in enhanced occurrence of cortical up-states with layer IV onset. During the first 50 ms of up-states, the evoked MUA was significantly stronger in all layers compared to the spontaneous up-states, presumably due to the synchronous activation of neuronal populations responsible for the generation of the transient evoked response to somatosensory stimulation. Besides, in the current source density (CSD) profile a new source appeared in layer V of evoked activity, which may represent the passive recurrent flow related to the strong synaptic activity in layer IV and layer III induced by sensory stimulation [74]. The i.p. delivery of ketamine (60 mg/kg induction) in combination with medetomidine (0.5 mg/kg induction) in adult rats has been used by Bettinardi and collaborators to study the effect of anesthesia levels on spontaneous LFPs recorded simultaneously in the infragranular layers of coupled (medial prefrontal mPFC and cingulate CC) and uncoupled (auditory A1 and second somatosensory S2) cortexes. Passing from deep (50 min after induction) to light (135 min after induction) levels, ketamine–medetomidine anesthesia was shown to induce a modulation in the oscillatory activity: in all areas a decrease in power at frequencies around the gamma band (30–50 Hz) was revealed. In cortical areas like mPF and CC, instead, which belong to the same functional network, an increase in power of frequencies around the alpha rhythm (8–15 Hz) and an increase of coupling in both frequency ranges passing from deep to light anesthesia states was revealed. Thus, coupled areas exhibit a clear net increase in coupling in these frequency ranges (8–15 Hz and 30–50 Hz) during the progression to lighter states of anesthesia; moreover, even during deeper stages of anesthesia, areas belonging to the same functional network correlate at specific frequencies more than areas that do not participate in the same network [75]. In conclusion, ketamine strongly affects glutamatergic transmission, by inhibiting NMDA receptors and favoring AMPA activation. At the system level, ketamine effects are strongly dependent on the level of anesthesia, leading to different modulation of neuronal synchronization across frequency ranges from the alpha to the gamma band and alteration of the thalamocortical sensory pathways.

#### 3.1.4. Dexmedetomidine

Dexmedetomidine, the active component of the racemic drug medetomidine, is a highly selective α2-AR agonist which produces dose-dependent sedation, anxiolysis and analgesia (involving spinal and supraspinal sites) without causing respiratory depression [76]. Agonists of α2-ARs are frequently used in veterinary anesthesia where they affect sedation, muscle relaxation and analgesia through effects on the CNS. The concentration of α2-ARs in the rat brain has been shown to be low in the cortex and thalamus and high in the lower brain regions. Dexmedetomidine inhibits noradrenergic neurons in the *locus coeruleus* causing a complete disruption of the signals from the ventrobasal thalamus to the cortex [77]. Studies on NG108-15 neuroblastoma/glioma cell line and on cerebellar granule neurons isolated from Wistar rat pups revealed a strong reduction in the amplitude of delayed rectifier K+ current, which was correlated with the concentration of dexmedetomidine. A similar effect was noticed on Na+ currents and action potentials amplitudes, while L-type Ca^2+^ currents were not affected [78]. On anesthetized rodents, the effect of dexmedetomidine on brain activity seems to be highly correlated to the administration route: Hayton and collaborators found that the amplitude of SEPs induced by electrical stimulation of the tibial nerve of adult Wistar rats and recorded with an electrode inserted subcutaneously at the scalp over the contralateral somatosensory cortex, was severely decreased by intramuscular administration of 0.3 mg/kg dexmedetomidine [79]. On the contrary, in adult Sprague-Dawley rats, Li and collaborators described a non-significant alteration of amplitude and latency of SEPs recorded from the frontal and parietal regions of the scalp during tibial nerve stimulations by venous infusion of the same total amount of dexmedetomidine [80]. A different effect, instead, was detected by i.v. delivery of dexmedetomidine in rats with chronically implanted electrode array on the auditory cortex: both sedation (1.33 μg/kg/min for 10 min followed by 0.27 μg/kg/min) and loss-of-consciousness (2.00 μg/kg/min for 10 min followed by 0.4–0.5 μg/kg/min) did decrease the spontaneous and evoked firing rates, with a concomitant and dose-dependent increase in the response latency [81]. In the barrel cortex of adult Sprague Dawley rats, dexmedetomidine delivered i.v. at four different doses (1.86, 3.75, 5.63 and 7.50 ng/mL) produced a concentration-dependent attenuation of the spectral power of high frequency thalamocortical rhythms (30–200 Hz), but with a weaker effect than other anesthetic agents like isoflurane and propofol [82]. Thus, at least by i.v. delivery, dexmedetomidine exhibits a capability to suppress cortical activity in the sensory areas.

#### 3.1.5. Medetomidine-Midazolam-Butorphanol (MMB)

The benzodiazepine midazolam exerts a sedative effect without providing analgesia. For this reason, it is commonly used in combination with other agents, usually the α2-AR agonist anesthetic medetomidine and the opioid analgesic butorphanol. In C57/BL6 mice, intraperitoneal administration of a single dose of MMB mixture (0.3 mg/kg medetomidine, 4 mg/kg midazolam, 5 mg/kg butorphanol) induced anesthesia in about 20–30 min, with no additional doses required. In Sprague-Dawley rats, the induction time required by a single dose of MMB mixture (0.15, 2 and 2.5 mg/kg body weight, respectively) delivered subcutaneously was around 10 min; instead, with intraperitoneal administration this time increased to 25 min and required one or two additional doses (⅕ of the induction). The time required to reach a surgical anesthesia, that is the total time of duration of anesthesia from the induction, was found to be twice-fold in the case of subcutaneous injection. Thus, MMB administered by s.c. injection exerted a more rapid, complete and stable anesthetic effect as compared to MMB administered by i.p. injection [83]. Osanai & Tateno (2016) compared the effect of the i.p. delivery of MMB and ketamine-xylazine (KX) on LFPs recorded with a 16-channels probe along A1 auditory cortex of adult Wistar rats: the change of the anesthetic during the recording session showed no effect in cortical layers II/III and IV. In layer V, instead, a firing rate increase was detected after the MMB → KX change, while the firing rate decreased after the KX → MMB change [84].

#### 3.1.6. Sodium Pentobarbital

Pentobarbital is an oxybarbiturate analog of barbituric acid, which can be used as a sedative–hypnotic, anesthetic, and anticonvulsant. Pentobarbital’s mechanism of action is similar to that of benzodiazepines and propofol, as GABA_A_ receptors are activated resulting in enhanced GABA binding and opening of chloride channels leading to cellular hyperpolarization within the central nervous system. Similar to all GABAA agonists, pentobarbital has little or no analgesic activity. Administration of pentobarbital produces dose-dependent sedation, hypnosis, muscle relaxation, and depression of the sensory cortex and reticular activating system. It also induces dose-dependent respiratory depression which may require respiratory assistance [85,86]. At higher doses, pentobarbital possesses anticonvulsant and hypotensive properties [86].

Spatiotemporal changes in neuronal activities of the primary sensorimotor cortex (SmI) in response to noxious laser heat stimulation applied to the mid-tail were analyzed in adult Long-Evans rats under 50 mg/kg sodium pentobarbital i.p. anesthesia, with respect to the awake animal. LFPs and SUs (Single Units) were recorded with a longitudinal and a vertical multielectrode array chronically implanted in the SmI. The amplitude of fast and slow components of the evoked LFPs in the awake rat was reduced after administration of the anesthetic, and the LFPs were limited to the region of SmI corresponding to the tail. The inhibitory effect of sodium pentobarbital was observed also on the SU activity in all the areas belonging to SmI. The vertical array implanted into the tail region of SmI allowed to demonstrate that both LFPs and SUs, which were recorded from all the channels in the awake animal, were strongly reduced by anesthesia in the supragranular layers, while a significant activity was still observed in the deeper layers [87].

#### 3.1.7. Propofol

Propofol is a rapid-acting, sedative and hypnotic intravenous agent used for induction and maintenance of general anesthesia. It is also associated with a quick and ‘smooth’ recovery, which distinguishes it from many of the more traditional anesthetic regimens. However, propofol is structurally unrelated to other hypnotic compounds. It induces hypnosis rapidly and reliably, and has additive or synergistic hypnotic effects with many other agents (like barbiturates, benzodiazepines, opioids and ketamine) commonly used in anesthesia [88].

Propofol affects the positive modulation of the inhibitory function of the GABA neurotransmission through the ligand-dependent GABA A receptors to produce its sedative/anesthetic effects [89]. In particular, it appears to slow down the closing time of the channel, also acting as a blocker of the sodium channels [89]. Hill-Venning et al., Krasowski et al., and Siegwart et al. demonstrated the propofol action at GABA(A) receptors by harboring a beta-subunit; The isoform of the beta-subunit has little influence upon the GABA-modulating action of propofol and the N265M mutation in the TM2 region of the GABA(A) receptor beta-3-subunit abolishes the actions of propofol [90,91,92]. These findings were later consolidated in vivo by the seminal landmark-paper by Jurd et al., showing that the propofol-anesthesia endpoints hypnosis and immobility are mediated via beta-3-containing GABA(A) receptors [22]. It has been suggested that the endogenous cannabinoid system may contribute significantly to the anesthetic action and unique properties of propofol [93]. The pharmacokinetic profile of propofol is characterised by a fast distribution from the blood into tissues, a rapid metabolic clearance from the blood and a slow return of the drug from the peripheral compartment. The interaction of these processes produces a rapid onset and a short duration of action. Propofol is rapidly and extensively distributed to well perfused tissue (including the brain), then to lean muscle and finally to fat tissue [94,95].

Propofol is an injectable short-acting drug which induces a decreased level of consciousness. It is commonly used in animal anesthesia during electrophysiological recording. EEG research demonstrated a prominent reduction in the brain’s information integration capacity at gamma wave band frequencies during propofol anesthesia [95,96].

Klostermann and collaborators [97] demonstrated that propofol preferentially diminished scalp recorded high frequency oscillations (HFO) of cortical somatosensory evoked potentials probably through its action on inhibitory GABAergic interneurons in cerebral cortex. HFO are generated by thalamocortical projection fibers and intracortical activities involving inhibitory interneurons or fast inhibitory postsynaptic potentials of pyramidal neurons. The latency of ERPs recorded over the somatosensory cortex of rats during whisker stimulation was strongly increased by a constant intravenous infusion of propofol, while the amplitudes of ERPs and their main band components were reduced [55]. In the rat auditory cortex, instead, spontaneous and stimuli-evoked spiking rates recorded by a chronically implanted electrode array were decreased by i.v. delivery of propofol at both sedation (450 μg/kg/min) and loss-of-consciousness (600–700 μg/kg/min) doses, respect to the awake rat. The evoked response latencies, however, were not significantly affected by propofol [81].

### 3.2. Volatile Anesthetics

Volatile anesthetics are a class of substances liquid at room temperature, but evaporating easily for administration by inhalation[12]. The majority of inhalation anesthetics for in vivo electrophysiological experiments are halogenated organic substances, which increase anesthetic potency while improving stability. Current knowledge about the mechanism of action of volatile anesthetics reveals that these molecules primarily affect GABA-ergic transmission and gap junction functionality. Their main protein targets are the tandem pore potassium channels, voltage-gated sodium channels, NMDA receptors, and the pentameric ligand-gated ion channels (pLGICs), including glycine receptors (GlyRs) and γ-aminobutyric acid receptors (GABAARs) [25]. An ideal volatile anesthetic agent should offer smooth and reliable induction and maintenance of general anesthesia, with minimal effects on brain functions and without altering electrophysiological recordings. Although none of the agents currently in use are ideal, many of them have some of the desirable properties, like isoflurane which is the most used anesthetic in laboratory animals [12,98].

#### 3.2.1. Isoflurane

Thanks to its shortest mean induction and recovery times, isoflurane is often used as induction anesthetic for surgical procedures, being subsequently substituted with other compounds for the recording session [99]. When used during experimental recording sessions, isoflurane was shown to affect the brain activity: SEPs induced by electrical stimulation of the tibial nerve of adult Wistar rats anesthetized with isoflurane (5% induction and 2% maintenance) and recorded with an electrode inserted subcutaneously at the scalp over the contralateral somatosensory cortex, revealed an increase in the latency and a decrease in the amplitude, compared to other anesthetics [79]. The same strong reduction in the amplitude was detected on ERPs recorded over the somatosensory cortex during whiskers stimulation [55]. In the auditory A1 cortex of rats, both spontaneous and stimuli-evoked firing rate recorded by a chronically implanted electrode array, was strongly reduced by sedation (0.4%) and loss-of-consciousness (0.8–1%) doses of isoflurane in comparison to the awake animal, while no effect was detected on response latency [81]. Conversely, higher doses of isoflurane from 1.4 to 2.2% were shown not only to reduce the number of active single units, but also to increase their response onset latency compared to the awake condition; the evoked LFPs were burst-suppressed and a typical pattern with alternating high amplitude bursts and silent periods emerged [100]. The level of isoflurane anesthesia proportionally affects the functional interaction between the cortex and the thalamus: from LFP recordings in adult Long-Evans rats chronically implanted with electrodes in the parietal lobe and thalamic region, it was shown that increasing the anesthesia level from light (1%) to deep (2.5%) caused an increase in the interactions between the two brain areas mainly in the alpha frequencies, and preferentially in the thalamus-to-cortex direction [101]. From EEG recordings on S1FL (forelimb) and S1BF (barrel field) primary somatosensory cortex areas in adult Sprague-Dawley rats, a burst suppression and a reduction in the delta (1–4 Hz) and gamma (>30 Hz) bands power appeared with deep isoflurane anesthesia concentration (1.8%), with respect to moderate and light concentrations (1.5 and 1.0%, respectively [102]). In the primary visual cortex (V1) of adult Long-Evans rats, four different concentrations of isoflurane ranging from very light (0.6–0.9%) to light (0.9–1.2%), middle (1.2–1.6%) and deep (1.6–2.0%) levels influenced both the spontaneous and the evoked multi-units (MUs) and LFPs: evoked firing rate was much more influenced by anesthesia with respect to the spontaneous one and tended to decrease proportionally with isoflurane concentration. The variability of neuronal responses in V1 strongly depended on the level of anesthesia, with very light and light concentrations providing a response variance similar to the awake state, and an increasing variability under middle and deep anesthesia. The power spectrum of spontaneous LFPs decreased significantly with increasing concentration [103]. Other researchers studied the effect of isoflurane anesthesia (at 0.5%, 0.75% and 1.0% doses), combined with a constant infusion of xylazine, on MUA and LFP recorded throughout all the layers of visual (V1) and prefrontal (PFC) cortexes of ferrets during visual or auditory stimulation [104]: compared with awake animals, increasing concentrations of the anesthetics altered the laminar distribution of MU firing in V1 cortex during 1 Hz visual stimulation and increased the relative strength of the response to visual stimulation, predominantly in layer IV. The LFP spectral power was driven by the temporal patterning of the visual stimulus and increasing concentrations of isoflurane-xylazine increased the power across all frequency bands, but with the greatest enhancement in the lower frequency bands and in granular (IV) and infragranular (V-VI) layers. In PFC, instead, the modulation of LFP observed mainly in layers II/III of awake animals during visual stimulation was lost in the anesthetized animal, thus demonstrating that isoflurane-xylazine alters the representation of sensory input in PFC. The anesthetic also altered the responses of V1 and PFC during an auditory stimulation, with loss of modulation of LFP and absence of MUA activity. Overall, these results showed that isoflurane-xylazine anesthesia did not suppress sensory responses in V1, but rather induced specific changes to the temporal structure of MUA and LFP response patterns and compromised the connectivity between V1 and higher cortical areas such as PFC. The specific increase in the visually evoked firing rate in V1 layer IV could be due to different expressions of xylazine molecular targets across cortical layers. Aasebo and collaborators compared the effect of Isoflurane 1.5% with other anesthesia combinations (isoflurane 1% + midazolam 1 mg/kg, ketamine 100 mg/kg + xylazine 5 mg/kg) and with the awake state in the V1 cortex of adult Long Evans rats by LFPs and spike recordings with chronically implanted tetrodes [105]. In general, the firing rate of stimulus-evoked responses decreased in the anesthesia regimens compared to the awake animal, with a major reduction for inhibitory neurons: this effect was mainly due to a reduction in the spontaneous firing rate and was prominent for isoflurane, while ketamine-xylazine did not produce significant difference with respect to the awake state. In the evoked LFPs and single units, the latencies of both the negative and the positive peaks were significantly longer in the anesthetized rats, and the average amplitude of the negative peak increased. By comparing pairs of neurons, in both evoked and spontaneous time periods higher correlations coefficients appeared in anesthesia with respect to awake, which were prominent in the case of isoflurane regimen. Only during isoflurane regimen, the coefficient of variation did not show any decrease respect to the awake, thus indicating that isoflurane maintained a regularity in the firing of the units similar to the awake condition [105]. The action of anesthetics on thalamic nuclei is different from cortical areas: isoflurane at three different doses (1.5, 2 and 2.5%) was shown to exert the same effect of infused medetomidine (0.1 and 0.3 mg/kg/h) on spontaneous LFPs recorded from the central medial (CM) and ventral posterolateral (VPL) thalamic nuclei of adult Wistar rats. Firing rates and LFP power progressively decreased in the CM with increasing isoflurane and medetomidine dosage, while no significant changes were observed in the VPL, thus indicating a specific effect of anesthesia on the thalamic CM [106].

#### 3.2.2. Sevoflurane

Sevoflurane is a halogenated ether anesthetic that is widely used in modern anesthesiology in humans, due to favorable clinical characteristics such as rapid pharmacokinetics and lack of airway irritability. In animals, a study was conducted in prefrontal (PFC) and parietal (PC) cortexes and in the central thalamus (CT) of adult Sprague-Dawley rats anesthetized with doses varying from 1.6% to 2.8% with 0.2% incremental doses: at low doses, sevoflurane increased beta/low gamma power of spontaneous LFPs in all the three areas, and also increased the beta/low gamma PFC-CT and bilateral PFC coherence. At doses causing loss of movements, the beta/low gamma coherence decreased followed by an increase in slow-delta power. At higher doses, a further increase in the slow-delta power was noticed, concomitant with a slow-delta coherence increase between the different areas [107].

Box 2 **Example of the effect of anesthesia on evoked LFPs in the rat somatosensory barrel cortex.**

**Figure 1:**
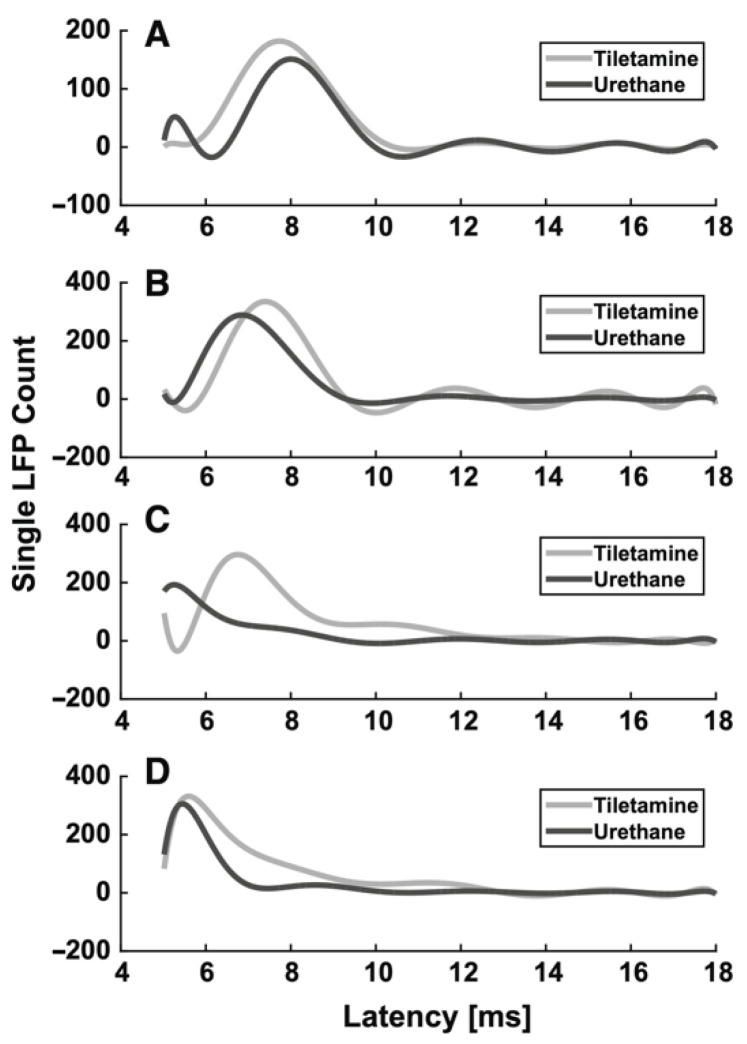
Histogram estimation of the response onset latency at different recording depths. (**A**) 320 µm (layer II). (**B**) 420 µm (layer III), (**C**) 720 µm (layer IV), (**D**) 920 µm (layer Va).

**Figure 2:**
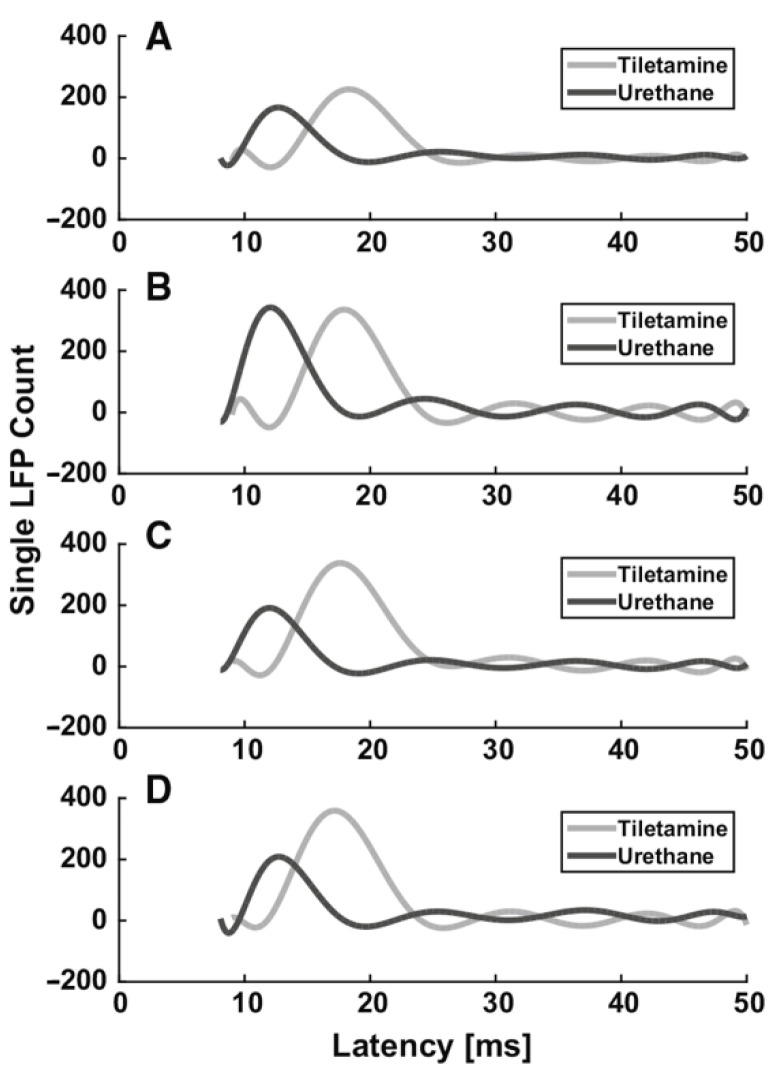
Histogram estimation of the response peak latency at different recording depths. (**A**) 320 µm (layer II). (**B**) 420 µm (layer III), (**C**) 720 µm (layer IV), (**D**) 920 µm (layer Va).

From the previous paragraphs it clearly emerges that the use of different anesthetics during in vivo electrophysiological recordings affects sensory-evoked LFPs. As an example, we summarize the different effects of two different anesthetics (tiletamine—which is a ketamine analog—combined with xylazine, and urethane) on whisker-evoked responses in the rat barrel cortex [108,109]. At each cortical depth in layers II, III, IV and Va, 500 single-trial responses were recorded through standard Ag/AgCl electrodes upon the principal whisker mechanical deflection. The effect of anesthesia on the sensory information processing was assessed by comparing the main features (i.e., latency of the response onset, latency of the principal peak and amplitude of the principal peak) of the evoked single LFPs recorded from different rats, anesthetized with either tiletamine-xylazine (*n* = 5) or urethane (*n* = 4). Histograms were made from the extracted main parameters of the LFP traces. To better illustrate the trends in each anesthetic, estimations of the histograms were calculated by fitting higher order polynomials (Figure 1 and Figure 2). Interestingly, we found that the onset and the peak latency of signals recorded under the two anesthetics followed two different distributions, accompanied by a layer-dependent behavior. Under tiletamine-xylazine, the evoked responses recorded from the superficial layers II and III showed less variability than those recorded from the deeper layers IV and Va. This was highlighted in the distributions of the onset (Figure 1, in gray) and peak (Figure 2, in gray) latencies: in particular, the distribution of the onset latencies was wider in the deeper layers than in the superficial ones. This trend was instead reversed in the evoked LFPs responses recorded under urethane: the distributions of the onset (Figure 1, in black) and peak (Figure 2, in black) latencies were slightly narrower in the deeper layers than in the superficial ones. By comparing the distributions obtained from the two different anesthetics, it seemed that the onset latency distributions were narrower in the case of urethane and that the peak of the evoked LFPs occurred earlier than the one recorded under tiletamine-xylazine anesthesia. In summary, evoked responses seemed to be deeply influenced by the type of anesthetic used, as urethane appeared to promote more precise, stronger and faster responses than those generated under tiletamine-xylazine.

## 4. Conclusions and Future Perspectives

General anesthesia is an induced, reversible and controlled loss of consciousness, required to perform surgery on both humans and laboratory animals. Although the mechanism of action of general anesthetics is not entirely clear, they are known to interrupt activity propagation along neuronal pathways, preventing the central nervous system from processing stimuli. The main desired consequence during surgical procedures is the state of unconsciousness and immobility. Experiments dealing with anesthetized animals, and particularly rodents, represent an important part of biomedical research, with the aim to understand the pathogenesis of human diseases and to develop effective and safe treatments. It is imperative that animal experimentation is conducted according to ethics attentive guidelines, preventing animal suffering and maintaining high quality standards. According to the Declaration of Bologna, signed in the “3rd World Congress on Alternatives and Animal Use in the Life Sciences” (1999), “humane science is a prerequisite for good science, and is best achieved in relation to laboratory animal procedures by the vigorous promotion and application of the Three Rs (Replacement of animal experiments; Reduction of the number of animals used; Refinement of procedures)”. Unfortunately, to date there are no published works that clarify how to select the most suitable anesthetic for different experimental measurements, with the purpose of limiting to the minimum the interference between the anesthetic and the electrophysiological recordings, and increasing result accuracy and reliability at the same time. Different types of anesthesia have different and specific effects on evoked and spontaneous activity: in this review, we summarized what is reported in literature on various brain areas, highlighting the main differences between the most commonly used anesthetics. Depending on the specific anesthetic, an alteration on frequency, latency and amplitude of spontaneous and/or evoked cortical activities may arise. In general, the amplitude is reduced and the latency is increased, but these effects are also strongly dependent on the dose and the route of administration. In general, the choice of the type of anesthesia needs to be defined by the type of experiment that has to be performed, considering both the animal welfare (absence, for instance, of pain and respiratory problems) and the impact on the recordings, according to the type of cortical area to study.

Among volatile anesthetics, isoflurane is the easiest to use, has less side effects and the level of anesthesia can be easily adjusted by controlling the gas concentration at the vaporizer. For what concerns injectable ones, medetomidine combined with midazolam proves to be effective and its effects are reversible, which is very useful in the case of chronic experiments. Also ketamine-xylazine mixture provides -good and stable levels of anesthesia inrodents, with the disadvantage that the inhibitory effect on NMDA receptors makes it not suitable for experiments where glutamatergic synaptic -plasticity is involved. Urethane, also pretty much used in rodents, better preserves glutamatergic plasticity but, also according to our own experience, induces a larger variability in evoked sensory responses. Moreover, keeping a constant level of anesthesia and physiological parameters such respiration and heart frequencies and body temperature is more difficult.

Experimental work still needs to be carried on in order to advance the knowledge of mechanisms of action of general anesthetics and their effects. Besides specific in vivo models, today a meaningful contribution could be given by in silico approaches that use experimentally and -omic fed databases to elucidate system biological networks. Like in other areas of network pharmacology, the application of a systems biology approach to better understand the complex biological connections affected by general anesthetics, could lead to a better and more advanced modeling of such complex and nonlinear systems. Although this is still an emerging field for anesthetics, examples of network pharmacology application to anesthetics have led to advanced models [110,111,112,113], thus showing the potential of these analytical tools empowering researchers with an overarching sight of molecular pathways elicited by general anesthetics.

**Table 1:** Summary of the main injectable and volatile anesthetics.

Name	Mechanism of Action	Start and Duration of Action	Use in Rodents	Use in Humans	Side Effects	Major Effects on Rodents Cortical Recordings	References
Injectable anesthetics							
Urethane	moderate GABAA, glycine and nACh receptors excitation, NR1A/NR2A NMDA and GluR1/GluR2 AMPA receptors inhibition	Start 1–5 min Duration hours	i.p. Induction: 1.5–2 g/kg body mass; additional doses: 10–20% of induction. i.v. 1.75 g/kg body mass	No	Mucus in the respiratory tract	Evoked activity: ↑ latency, ↓ SEP and VSDI amplitude (dose-dependent effect) Spontaneous activity: ↓ burst and firing rates, ↑ burst duration, ↑ number of spikes in a burst (dose-dependent effect)	[49]
Ketamine	NMDA channel antagonist; AMPA receptors upregulator and µ-type opioid receptors agonist; anesthesia, analgesia, amnesia, sedation	Start 1–5 min. Duration 15 min	Combined with xylazine/medetomidine. i.p. Induction: 40–100 mg/kg body mass; additional doses: 10–30% of induction	Yes	Laryngospasm, hypertension, tachycardia, hypersalivation, vomiting, psychiatric symptoms	Alone Evoked activity: ↑ LFP amplitude, ↑ long lasting LFP (compared to awake) With Xylazine: Evoked activity: LIV onset; Spontaneous activity: LV onset; ↓ frequency, ↑ amplitude (dose-dependent effect)	[114]
Xylazine	alpha2 adrenoceptor agonist; sedation, muscle relaxation, analgesia	Start 1–5 min. Duration 15 min	Combined with ketamine. i.p. Induction: 5–15 mg/kg body mass; additional doses: 30% of induction	No	In humans, it could cause central nervous system depression, respiratory depression, bradycardia,	see Ketamine	[115]
Medetomidine/Dexmedetomidine	alpha2 adrenoceptor agonist; sedation, anxiolysis, analgesia	Start: Combined with midazolam/butorphanol: Start: i.p. 25 min, s.c. 10 min	Combined with ketamine. i.p. Induction 0.5 mg/kg body mass Combined with midazolam/butorphanol. i.p. or s.c. 0.15–0.3 mg/kg body mass; additional doses (if needed for i.p.): 20% of induction	Yes ^1^		Alone: Evoked activity: ↑ latency, ↓ amplitude, ↓ firing rates (dose/route-dependent effect), weak ↓ power of HF thalamocortical activity (dose-dependent) With Midazolam/Butorphanol: Spontaneous activity: ↓ LV firing rate	[116]
Midazolam	GABA receptor positive allosteric modulator; anterograde, anxiolytic, sedation, hypnosis amnesia	Start: i.p. 25 min, s.c. 10 min combined with medetomidine/butorphanol	Combined with medetomidine/butorphanol. i.p. or s.c. Induction: 2–4 mg/kg body mass; additional doses (if needed for i.p.): 20% of induction	Yes	Respiratory depression, hypotension, agitation	see Medetomidine	[117]
Butorphanol	opioid; analgesia	Start: i.p. 25 min, s.c. 10 min combined with medetomidine/midazolam Duration: 45–60 min combined with medetomidine/midazolam	Combined with medetomidine/midazolam. i.p. or s.c. Induction: 2.5–5 mg/kg body mass; additional doses (if needed for i.p.): 20% of induction	Yes ^2^		see Medetomidine	[118,119]
Sodium pentobarbital	GABAA receptor agonist	Duration 120 min	i.p. Induction: 50 mg/kg body mass; additional doses not required	Yes ^3^		Evoked activity: ↓ LFP and SU amplitude	[120]
Propofol	GABA positive allosteric modulator; sedation, hypnosis, amnesia		i.v. 450–700 μg/kg/min	Yes	Respiratory depression, apnea, hypotension, painful injection	Evoked activity: ↑ latency, ↓ amplitude Spontaneous activity: ↓ high frequency oscillations	[121]
Volatile anesthetics							
Isoflurane	tandem pore K channels, Nav channels, NMDA, glycine and GABAA receptors agonist	Start 10 min, Duration: 5 min	Inhalation. Induction: 2–5%; maintenance: 1.5–2.5%	Yes	Respiratory depression, irregular heartbeat, low blood pressure	Evoked activity: ↑ latency, ↓ amplitude, ↓ firing rate (>variability of neuronal responses) (dose-dependent effect). Spontaneous activity: ↓ firing rate, ↓ delta-gamma bands (dose-dependent effect)	[122]
Sevoflurane	GABAA. glycine potentiation, NMDA and nACh inhibition		Inhalation. Induction: 1.6–2.8%	Yes	Agitation, bradycardia, hypotension, cough, vomiting	Spontaneous activity: ↓ beta-gamma coherence, ↑ slow delta power, ↑ delta coherence (dose-dependent effect)	[123]

^1^ Dexmedetomidine is used into clinical practice as a short-term sedative (<24 h) and sedation in the intensive care unit (ICU). ^2^ It may be used as a supplement for balanced general anesthesia. Butorphanol is also quite effective at reducing post-operative shivering. ^3^ It has been used as a sedative and hypnotic in the short-term management of insomnia. It has also been used for premedication in anesthetic procedures.

## Data Availability

The datasets generated during and/or analysed during the current study are available from the corresponding author S.V. on reasonable request

## References

[1] Brown E.N., Lydic R., Schiff N.D. (2010). General anesthesia, sleep, and coma. N. Engl. J. Med..

[2] Kurdi M.S., Ramaswamy A.H. (2015). Anesthetizing animals: Similar to humans yet, peculiar?. Anesth. Essays Res..

[3] Mashour G.A., Alkire M.T. (2013). Evolution of consciousness: Phylogeny, ontogeny, and emergence from general anesthesia. Proc. Natl. Acad. Sci. USA.

[4] Campoy L., Read M. (2013). Small Animal Regional Anesthesia and Analgesia.

[5] Gargiulo S., Greco A., Gramanzini M., Esposito S., Affuso A., Brunetti A., Vesce G. (2012). Mice anesthesia, analgesia, and care, Part I: Anesthetic considerations in preclinical research. ILAR J..

[6] Chou R., Gordon D.B., de Leon-Casasola O.A., Rosenberg J.M., Bickler S., Brennan T., Carter T., Cassidy C.L., Chittenden E.H., Degenhardt E. (2016). Management of postoperative pain: A clinical practice guideline from the American pain society, the American Society of Regional Anesthesia and Pain Medicine, and the American Society of Anesthesiologists’ committee on regional anesthesia, executive committee, and administrative council. J. Pain.

[7] Dawkins M. (2012). Animal Suffering: The Science of Animal Welfare.

[8] Flecknell P.A., Thomas A.A. (2015). Comparative anesthesia and analgesia of laboratory animals. Vet. Anesth. Analg..

[9] Huang Z., Wang Z., Zhang J., Dai R., Wu J., Li Y., Liang W., Mao Y., Yang Z., Holland G. (2014). Altered temporal variance and neural synchronization of spontaneous brain activity in anesthesia. Hum. Brain Mapp..

[10] Hohlbaum K., Bert B., Dietze S., Palme R., Fink H., Thöne-Reineke C. (2018). Impact of repeated anesthesia with ketamine and xylazine on the well-being of C57BL/6JRj mice. PLoS ONE.

[11] Garcia-Pereira F. (2018). Epidural anesthesia and analgesia in small animal practice: An update. Vet. J..

[12] Clar D.T., Richards J.R. (2020). Anesthetic Gases. StatPearls [Internet].

[13] Wolfe R.C. (2020). Inhaled anesthetic agents. J. Perianesthesia Nurs..

[14] Billard V. (2015). Pharmacokinetic-pharmacodynamic relationship of anesthetic drugs: From modeling to clinical use. F1000Research.

[15] Brown E.N., Pavone K.J., Naranjo M. (2018). Multimodal general anesthesia: Theory and practice. Anesth. Analg..

[16] Field R.R. (2018). The syringe driver: Continuous subcutaneous infusions in palliative care. Anesth. Analg..

[17] Meyer R.E., Fish R. (2008). Pharmacology of injectable anesthetics, sedatives, and tranquilizers. Anesthesia and Analgesia in Laboratory Animals.

[18] Miller A.L., Theodore D., Widrich J. (2020). Inhalational Anesthetic. StatPearls [Internet].

[19] Berry S.H. (2015). Injectable anesthetics. Vet. Anesth. Analg..

[20] Buitrago S., Martin T.E., Tetens-Woodring J., Belicha-Villanueva A., Wilding G.E. (2008). Safety and efficacy of various combinations of injectable anesthetics in BALB/c mice. J. Am. Assoc. Lab. Anim. Sci..

[21] Posner L.P., Burns P. (2009). Injectable anaesthetic agents. Vet. Pharmacol. Ther..

[22] Jurd R., Arrasa M., Lambert S., Drexler B., Siegwart R., Crestani F., Zaugg M., Vogt K.E., Ledermann B., Antkowiak B. (2003). General anesthetic actions in vivo strongly attenuated by a point mutation in the GABAA receptor β3 subunit. Faseb J..

[23] Fish R.E. (1997). Pharmacology of injectable anesthetics. Anesthesia and Analgesia in Laboratory Animals.

[24] Forman S.A., Ishizawa Y. (2015). Inhaled Anesthetic Pharmacokinetics: Uptake, Distribution, Metabolism, and toxicity. Miller’s Anesthesia.

[25] Campagna J.A., Miller K.W., Forman S.A. (2003). Mechanisms of actions of inhaled anesthetics. N. Engl. J. Med..

[26] Smith R.A., Porter E.G., Miller K.W. (1981). The solubility of anesthetic gases in lipid bilayers. Biochim. Biophys. Acta (BBA)—Biomembr..

[27] Behne M., Wilke H.-J., Harder S. (1999). Clinical pharmacokinetics of sevoflurane. Clin. Pharmacokinet..

[28] Schwarzkopf K., Schreiber T., Preussler N.-P., Gaser E., Hüter L., Bauer R., Schubert H., Karzai W. (2003). Lung perfusion, shunt fraction, and oxygenation during one-lung ventilation in pigs: The effects of desflurane, isoflurane, and propofol. J. Cardiothorac. Vasc. Anesth..

[29] Urban B. (2008). The site of anesthetic action. Modern Anesthetics.

[30] Grasshoff C., Rudolph U., Antkowiak B. (2005). Molecular and systemic mechanisms of general anesthesia: The ‘multi-site and multiple mechanisms’ concept. Curr. Opin. Anesthesiol..

[31] Alkire M.T., Hudetz A.G., Tononi G. (2008). Consciousness and anesthesia. Science.

[32] Rudolph U., Antkowiak B. (2004). Molecular and neuronal substrates for general anaesthetics. Nat. Rev. Neurosci..

[33] Kelz M.B., Mashour G.A. (2019). The biology of general anesthesia from paramecium to primate. Curr. Biol..

[34] Li D., Vlisides P.E., Kelz M.B., Avidan M.S., Mashour G.A. (2019). Dynamic cortical connectivity during general anesthesia in healthy volunteers. Anesthesiology.

[35] Kitamura A., Marszalec W., Yeh J.Z., Narahashi T. (2003). Effects of halothane and propofol on excitatory and inhibitory synaptic transmission in rat cortical neurons. J. Pharmacol. Exp. Ther..

[36] Franks N., Lieb W. (1994). Molecular and cellular mechanisms of general anesthesia. Nature.

[37] De Sousa S.L., Dickinson R., Lieb W.R., Franks N.P. (2000). Contrasting synaptic actions of the inhalational general anesthetics isoflurane and xenon. Anesth. J. Am. Soc. Anesth..

[38] Urban B.W., Bleckwenn M., Barann M. (2006). Interactions of anesthetics with their targets: Non-specific, specific or both?. Pharmacol. Ther..

[39] Forman S.A., Miller K.W. (2016). Mapping general anesthetic sites in heteromeric gamma-aminobutyric acid type a receptors reveals a potential for targeting receptor subtypes. Anesth. Analg..

[40] Hemmings H.C., Riegelhaupt P.M., Kelz M.B., Solt K., Eckenhoff R.G., Orser B.A., Goldstein P.A. (2019). Towards a comprehensive understanding of anesthetic mechanisms of action: A decade of discovery. Trends Pharmacol. Sci..

[41] Fourati Z., Howard R.J., Heusser S.A., Hu H., Ruza R.R., Sauguet L., Lindahl E., Delarue M. (2018). Structural basis for a bimodal allosteric mechanism of general anesthetic modulation in pentameric ligand-gated ion channels. Cell Rep..

[42] Minami K., Uezono Y. (2006). Gq protein-coupled receptors as targets for anesthetics. Curr. Pharm. Des..

[43] Pavel M.A., Petersen E.N., Wang H., Lerner R.A., Hansen S.B. (2020). Studies on the mechanism of general anesthesia. Proc. Natl. Acad. Sci. USA.

[44] Sloan T.B. (2002). Anesthetics and the brain. Anesthesiol. Clin. N. Am..

[45] Voss L.J., Sleigh J.W. (2020). A metabolic mechanism for anaesthetic suppression of cortical synaptic function in mouse brain slices—A pilot investigation. Int. J. Mol. Sci..

[46] Laredo F. (2015). Injectable Anesthetics.

[47] Hara K., Harris R.A. (2002). The anesthetic mechanism of urethane: The effects on neurotransmitter-gated ion channels. Anesth. Analg..

[48] Simons D.J., Carvell G.E., Hershey A.E., Bryant D.P. (1992). Responses of barrel cortex neurons in awake rats and effects of urethane anesthesia. Exp. Brain Res..

[49] Pagliardini S., Funk G.D., Dickson C.T. (2013). Breathing and brain state: Urethane anesthesia as a model for natural sleep. Respir. Physiol. Neurobiol..

[50] Maggi C., Meli A. (1986). Suitability of urethane anesthesia for physiopharmacological investigations in various systems Part 1: General considerations. Experientia.

[51] Koblin D.D. (2002). Urethane: Help or hindrance?. Anesth. Anelgesia.

[52] Sceniak M.P., MacIver M.B. (2006). Cellular actions of urethane on rat visual cortical neurons in vitro. J. Neurophysiol..

[53] Reyes-Puerta V., Yang J.-W., Siwek M.E., Kilb W., Sun J.-J., Luhmann H.J. (2016). Propagation of spontaneous slow-wave activity across columns and layers of the adult rat barrel cortex in vivo. Brain Struct. Funct..

[54] Clement E.A., Richard A., Thwaites M., Ailon J., Peters S., Dickson C.T. (2008). Cyclic and sleep-like spontaneous alternations of brain state under urethane anesthesia. PLoS ONE.

[55] Rojas M.J., Navas J.A., Rector D.M. (2006). Evoked response potential markers for anesthetic and behavioral states. Am. J. Physiol. Regul. Integr. Comp. Physiol..

[56] Hama N., Ito S.-I., Hirota A. (2015). Optical imaging of the propagation patterns of neural responses in the rat sensory cortex: Comparison under two different anesthetic conditions. Neuroscience.

[57] Unichenko P., Yang J.-W., Kirischuk S., Kolbaev S., Kilb W., Hammer M., Krueger-Burg D., Brose N., Luhmann H.J. (2018). Autism related neuroligin-4 knockout impairs intracortical processing but not sensory inputs in mouse barrel cortex. Cereb. Cortex.

[58] Erchova I.A., Lebedev M.A., Diamond M.E. (2002). Somatosensory cortical neuronal population activity across states of anesthesia. Eur. J. Neurosci..

[59] Devonshire I.M., Grandy T.H., Dommett E.J., Greenfield S.A. (2010). Effects of urethane anesthesia on sensory processing in the rat barrel cortex revealed by combined optical imaging and electrophysiology. Eur. J. Neurosci..

[60] Sha O., Hao Y., Cho E., Zhou L. (2015). Clinical Applications and Side Effects of Ketamine. Ketamine: Use and Abuse.

[61] Kalmoe M.C., Janski A.M., Zorumski C.F., Nagele P., Palanca B.J., Conway C.R. (2020). Ketamine and nitrous oxide: The evolution of NMDA receptor antagonists as antidepressant agents. J. Neurol. Sci..

[62] Zanos P., Moaddel R., Morris P.J., Georgiou P., Fischell J., Elmer G.I., Alkondon M., Yuan P., Pribut H.J., Singh N.S. (2016). NMDAR inhibition-independent antidepressant actions of ketamine metabolites. Nature.

[63] Hirota K., Kubota T., Ishihara H., Matsuki A. (1999). The effects of nitrous oxide and ketamine on the bispectral index and 95% spectral edge frequency during propofol–fentanyl anesthesia. Eur. J. Anaesthesiol..

[64] Morgan C.J., Curran H.V., Drugs I.S.C.o. (2012). Ketamine use: A review. Addiction.

[65] Kurdi M.S., Theerth K.A., Deva R.S. (2014). Ketamine: Current applications in anesthesia, pain, and critical care. Anesth. Essays Res..

[66] Dimitriadis G., Fransen A.M., Maris E. (2014). Sensory and cognitive neurophysiology in rats. Part 2: Validation and demonstration. J. Neurosci. Methods.

[67] Dimitriadis G., Fransen A.M., Maris E. (2014). Sensory and cognitive neurophysiology in rats, Part 1: Controlled tactile stimulation and micro-ECoG recordings in freely moving animals. J. Neurosci. Methods.

[68] Struck M.B., Andrutis K.A., Ramirez H.E., Battles A.H. (2011). Effect of a short-term fast on ketamine–xylazine anesthesia in rats. J. Am. Assoc. Lab. Anim. Sci..

[69] Wixson S., White W., Hughes Jr H., Lang C., Marshall W. (1987). A comparison of pentobarbital, fentanyl-droperidol, ketamine-xylazine and ketamine-diazepam anesthesia in adult male rats. Lab. Anim. Sci..

[70] Van Pelt L. (1977). Ketamine and xylazine for surgical anesthesia in rats. J. Am. Vet. Med Assoc..

[71] Fontanini A., Bower J.M. (2005). Variable coupling between olfactory system activity and respiration in ketamine/xylazine anesthetized rats. J. Neurophysiol..

[72] Tandon S., Kambi N., Jain N. (2008). Overlapping representations of the neck and whiskers in the rat motor cortex revealed by mapping at different anaesthetic depths. Eur. J. Neurosci..

[73] Ordek G., Groth J.D., Sahin M. (2013). Differential effects of ketamine/xylazine anesthesia on the cerebral and cerebellar cortical activities in the rat. J. Neurophysiol..

[74] Fiáth R., Kerekes B.P., Wittner L., Tóth K., Beregszászi P., Horváth D., Ulbert I. (2016). Laminar analysis of the slow wave activity in the somatosensory cortex of anesthetized rats. Eur. J. Neurosci..

[75] Bettinardi R.G., Tort-Colet N., Ruiz-Mejias M., Sanchez-Vives M.V., Deco G. (2015). Gradual emergence of spontaneous correlated brain activity during fading of general anesthesia in rats: Evidences from fMRI and local field potentials. Neuroimage.

[76] Naaz S., Ozair E. (2014). Dexmedetomidine in current anesthesia practice-a review. J. Clin. Diagn. Res. JCDR.

[77] Angel A. (1993). Central neuronal pathways and the process of anesthesia. BJA Br. J. Anaesth..

[78] Chen B.-S., Peng H., Wu S.-N. (2009). Dexmedetomidine, an α2-adrenergic agonist, inhibits neuronal delayed-rectifier potassium current and sodium current. Br. J. Anaesth..

[79] Hayton S., Kriss A., Muller D. (1999). Comparison of the effects of four anaesthetic agents on somatosensory evoked potentials in the rat. Lab. Anim..

[80] Li B.-H., Lohmann J.S., Schuler H.G., Cronin A.J. (2003). Preservation of the cortical somatosensory-evoked potential during dexmedetomidine infusion in rats. Anesth. Analg..

[81] Banks M., Moran N., Krause B., Grady S., Uhlrich D., Manning K. (2018). Altered stimulus representation in rat auditory cortex is not causal for loss of consciousness under general anesthesia. Br. J. Anaesth..

[82] Plourde G., Arseneau F. (2017). Attenuation of high-frequency (30–200 Hz) thalamocortical EEG rhythms as correlate of anaesthetic action: Evidence from dexmedetomidine. BJA: Br. J. Anaesth..

[83] Shibuta H., Yamana R., Kashimoto J., Kamio K., Suda A. (2019). Comparison of the anesthetic effect by the injection route of mixed anesthesia (medetomidine, midazolam and butorphanol) and the effect of this anesthetic agent on the respiratory function. J. Vet. Med Sci..

[84] Osanai H., Tateno T. (2016). Neural response differences in the rat primary auditory cortex under anesthesia with ketamine versus the mixture of medetomidine, midazolam and butorphanol. Hear. Res..

[85] Peters J.A., Kirkness E.F., Callachan H., Lambert J.J., Turner A.J. (1988). Modulation of the GABAA receptor by depressant barbiturates and pregnane steroids. Br. J. Pharmacol..

[86] Johnson A.B., Sadiq N.M. (2020). Pentobarbital. StatPearls [Internet].

[87] Kuo C.-C., Lee J.-C., Chiou R.-J., Tsai M.-L., Yen C.-T. (2015). Spatiotemporal changes of neuronal responses in the primary somatosensory cortex to Noxious tail stimulation in awake and pentobarbital-anesthetized rats. Chin. J. Physiol..

[88] Belrose J.C., Noppens R.R. (2019). Anesthesiology and cognitive impairment: A narrative review of current clinical literature. BMC Anesthesiol..

[89] Vanlersberghe C., Camu F. (2008). Propofol. Modern Anesthetics.

[90] Hill-Venning C., Belelli D., Peters J.A., Lambert J.J. (1997). Subunit-dependent interaction of the general anaesthetic etomidate with the γ-aminobutyric acid type A receptor. Br. J. Pharmacol..

[91] Krasowski M.D., Koltchine V.V., Rick C.E., Ye Q., Finn S.E., Harrison N.L. (1998). Propofol and other intravenous anesthetics have sites of action on the γ-aminobutyric acid type A receptor distinct from that for isoflurane. Mol. Pharmacol..

[92] Siegwart R., Jurd R., Rudolph U. (2002). Molecular determinants for the action of general anesthetics at recombinant α2β3γ2γ-aminobutyric acidA receptors. J. Neurochem..

[93] Fowler C.J. (2004). Possible involvement of the endocannabinoid system in the actions of three clinically used drugs. Trends Pharmacol. Sci..

[94] Shafer A., Doze V.A., Shafer S.L., White P.F. (1988). Pharmacokinetics and pharmacodynamics of propofol infusions during general anesthesia. Anesthesiol. J. Am. Soc. Anesthesiol..

[95] Purdon P., Pavone K., Akeju O., Smith A., Sampson A., Lee J., Zhou D., Solt K., Brown E. (2015). The ageing brain: Age-dependent changes in the electroencephalogram during propofol and sevoflurane general anesthesia. Br. J. Anaesth..

[96] Sahinovic M.M., Struys M.M., Absalom A.R. (2018). Clinical pharmacokinetics and pharmacodynamics of propofol. Clin. Pharmacokinet..

[97] Klostermann F., Funk T., Vesper J., Siedenberg R., Curio G. (2000). Propofol narcosis dissociates human intrathalamic and cortical high-frequency (>400 Hz) SEP components. Neuroreport.

[98] Landoni G., Lomivorotov V.V., Nigro Neto C., Monaco F., Pasyuga V.V., Bradic N., Lembo R., Gazivoda G., Likhvantsev V.V., Lei C. (2019). Volatile anesthetics versus total intravenous anesthesia for cardiac surgery. N. Engl. J. Med..

[99] Airaksinen A.M., Hekmatyar S.K., Jerome N., Niskanen J.P., Huttunen J.K., Pitkänen A., Kauppinen R.A., Gröhn O.H. (2012). Simultaneous BOLD fMRI and local field potential measurements during kainic acid–induced seizures. Epilepsia.

[100] Noda T., Takahashi H. (2015). Anesthetic effects of isoflurane on the tonotopic map and neuronal population activity in the rat auditory cortex. Eur. J. Neurosci..

[101] Cho D., Shin T.J., Ham J., Choi D.-H., Kim S., Jeong S., Kim H.-I., Kim J.G., Lee B. (2017). Differential modulation of thalamo-parietal interactions by varying depths of isoflurane anesthesia. PLoS ONE.

[102] Liu X., Zhu X.-H., Zhang Y., Chen W. (2013). The change of functional connectivity specificity in rats under various anesthesia levels and its neural origin. Brain Topogr..

[103] White B., Abbott L.F., Fiser J. (2012). Suppression of cortical neural variability is stimulus-and state-dependent. J. Neurophysiol..

[104] Sellers K.K., Bennett D.V., Hutt A., Williams J.H., Fröhlich F. (2015). Awake vs. anesthetized: Layer-specific sensory processing in visual cortex and functional connectivity between cortical areas. J. Neurophysiol..

[105] Aasebø I.E., Lepperød M.E., Stavrinou M., Nøkkevangen S., Einevoll G., Hafting T., Fyhn M. (2017). Temporal processing in the visual cortex of the awake and anesthetized rat. eNeuro.

[106] Abe Y., Tsurugizawa T., Le Bihan D. (2017). Water diffusion closely reveals neural activity status in rat brain loci affected by anesthesia. PLoS Biol..

[107] Guidera J.A., Taylor N.E., Lee J.T., Vlasov K.Y., Pei J., Stephen E.P., Mayo J.P., Brown E.N., Solt K. (2017). Sevoflurane induces coherent slow-delta oscillations in rats. Front. Neural Circuits.

[108] Cecchetto C., Mahmud M., Vassanelli S. Anesthesia effect on single local field potentials variability in rat barrel cortex: Preliminary results. Proceedings of the 37th Annual International Conference of the IEEE Engineering in Medicine and Biology Society (EMBC).

[109] Mahmud M., Cecchetto C., Vassanelli S. (2016). An automated method for characterization of evoked single-trial local field potentials recorded from rat barrel cortex under mechanical whisker stimulation. Cogn. Comput..

[110] Sanders R., Brian D., Maze M. (2008). G-Protein-Coupled Receptors. Modern Anesthetics.

[111] Wang C., Slikker W. (2008). Strategies and experimental models for evaluating anesthetics: Effects on the developing nervous system. Anesth. Analg..

[112] Colon E., Bittner E.A., Kussman B., McCann M.E., Soriano S., Borsook D. (2017). Anesthesia, brain changes, and behavior: Insights from neural systems biology. Prog. Neurobiol..

[113] Sun L.-H., Fan Y.-Y., Wang X., Zheng H.-B. (2020). Pharmacodynamic elucidation of glutamate & dopamine in ketamine-induced anesthesia. Chem. Biol. Interact..

[114] Nowacka A., Borczyk M. (2019). Ketamine applications beyond anesthesia—A literature review. Eur. J. Pharmacol..

[115] Ruiz-Colón K., Chavez-Arias C., Díaz-Alcalá J.E., Martínez M.A. (2014). Xylazine intoxication in humans and its importance as an emerging adulterant in abused drugs: A comprehensive review of the literature. Forensic Sci. Int..

[116] Gertler R., Brown H.C., Mitchell D.H., Silvius E.N. (2001). Dexmedetomidine: A novel sedative-analgesic agent. Bayl. Univ. Med Cent. Proc..

[117] Sivaramakrishnan G., Sridharan K. (2017). Nitrous oxide and midazolam sedation: A systematic review and meta-analysis. Anesth. Prog..

[118] Bansal P., Jain G. (2011). Control of shivering with clonidine, butorphanol, and tramadol under spinal anesthesia: A comparative study. Local Reg. Anesth..

[119] Kirihara Y., Takechi M., Kurosaki K., Kobayashi Y., Saito Y., Takeuchi T. (2016). Effects of an anesthetic mixture of medetomidine, midazolam, and butorphanol in rats—Strain difference and antagonism by atipamezole. Exp. Anim..

[120] Maldifassi M.C., Baur R., Sigel E. (2016). Functional sites involved in modulation of the GABAA receptor channel by the intravenous anesthetics propofol, etomidate and pentobarbital. Neuropharmacology.

[121] Bryson H.M., Fulton B.R., Faulds D. (1995). Propofol. Drugs.

[122] Soltanizadeh S., Degett T.H., Gögenur I. (2017). Outcomes of cancer surgery after inhalational and intravenous anesthesia: A systematic review. J. Clin. Anesth..

[123] Brioni J.D., Varughese S., Ahmed R., Bein B. (2017). A clinical review of inhalation anesthesia with sevoflurane: From early research to emerging topics. J. Anesth..

